# Supplementary material for: Comparative genomics provides new insights into the diversity, physiology, and sexuality of the only industrially exploited tremellomycete: Phaffia rhodozyma
Source: BMC Genomics. 2016 Nov 9;17:901. doi: 10.1186/s12864-016-3244-7 (PMC5103461; doi:10.1186/s12864-016-3244-7)
Supplement: Additional file 6: — List of orphan genes with links to PFAM (related to Additional file 1: Table S1). (ZIP 1428 kb) [file 12864_2016_3244_MOESM6_ESM.zip › BLAST_HTML_FTR/G00297_P.html]

BLAST Search Results


```
BLASTP 2.2.27+


Reference:
Stephen F. Altschul, Thomas L. Madden, Alejandro A. Schäffer,
Jinghui Zhang, Zheng Zhang, Webb Miller, and David J. Lipman (1997),
"Gapped BLAST and PSI-BLAST: a new generation of protein database
search programs", Nucleic Acids Res. 25:3389-3402.


Reference for
composition-based statistics:
Alejandro A. Schäffer, L. Aravind, Thomas L. Madden, Sergei
Shavirin, John L. Spouge, Yuri I. Wolf, Eugene V. Koonin, and
Stephen F. Altschul (2001), "Improving the accuracy of PSI-BLAST
protein database searches with composition-based statistics and
other refinements", Nucleic Acids Res. 29:2994-3005.


Database: nr
           71,551,133 sequences; 26,053,659,533 total letters


Query= G00297_P

Length=275
                                                                      Score     E
Sequences producing significant alignments:                          (Bits)  Value

emb|CDZ98239.1|  hypothetical protein [Xanthophyllomyces dendrorh...   467    4e-163
gb|ETS64000.1|  hypothetical protein PaG_02336 [Pseudozyma aphidi...  48.9    0.003 
dbj|GAC74011.1|  hypothetical protein PANT_9c00408 [Pseudozyma an...  48.1    0.005 
gb|KIY49436.1|  hypothetical protein FISHEDRAFT_41153 [Fistulina ...  45.8    0.027 
emb|CBQ68180.1|  conserved hypothetical protein [Sporisorium reil...  45.8    0.029 
gb|KNZ77176.1|  hypothetical protein J132_06171 [Termitomyces sp....  45.4    0.036 
dbj|GAK61885.1|  conserved hypothetical protein [Pseudozyma antar...  45.1    0.060 
gb|EST06686.1|  hypothetical protein PSEUBRA_SCAF3g04206 [Pseudoz...  44.7    0.076 
gb|EUC66862.1|  flavin containing monooxygenase FMO GS-OX3 [Rhizo...  44.3    0.087 
ref|XP_007315756.1|  hypothetical protein SERLADRAFT_446905 [Serp...  43.5    0.14  
emb|CDS01462.1|  hypothetical protein [Sporisorium scitamineum]       43.9    0.14  
gb|EGO02042.1|  hypothetical protein SERLA73DRAFT_166545 [Serpula...  43.5    0.15  
ref|XP_007268209.1|  hypothetical protein FOMMEDRAFT_147624, part...  43.5    0.15  
ref|XP_013240274.1|  hypothetical protein K437DRAFT_259903 [Tille...  43.5    0.17  
gb|KDQ64378.1|  hypothetical protein JAAARDRAFT_166529 [Jaapia ar...  43.1    0.21  
ref|XP_012193108.1|  hypothetical protein PHSY_007123 [Pseudozyma...  43.1    0.26  
ref|XP_002911173.1|  hypothetical protein CC1G_14604 [Coprinopsis...  42.7    0.28  
ref|XP_011386534.1|  hypothetical protein UMAG_00755 [Ustilago ma...  42.7    0.29  
ref|XP_007879121.1|  hypothetical protein PFL1_03413 [Pseudozyma ...  42.7    0.29  
ref|XP_007768011.1|  hypothetical protein CONPUDRAFT_164918 [Coni...  42.0    0.45  
emb|CUA76679.1|  hypothetical protein RSOLAG22IIIB_02146 [Rhizoct...  41.6    0.65  
gb|KIM68921.1|  hypothetical protein SCLCIDRAFT_1209138 [Sclerode...  41.6    0.66  
emb|CCF51250.1|  uncharacterized protein UHOR_01156 [Ustilago hor...  41.2    1.0   
gb|KDE09189.1|  hypothetical protein MVLG_00511 [Microbotryum vio...  40.8    1.2   
emb|CDI51690.1|  putative protein [Melanopsichium pennsylvanicum 4]   40.4    1.5   
gb|EMS23907.1|  peroxisomal membrane anchor protein [Rhodosporidi...  40.0    1.7   
emb|CCO26838.1|  Flavin-containing monooxygenase FMO GS-OX3 [Rhiz...  40.0    2.1   
emb|CEL57668.1|  hypothetical protein RSOLAG1IB_02411 [Rhizoctoni...  39.3    2.8   
emb|CDY65871.1|  BnaAnng21200D [Brassica napus]                       39.3    4.1   
gb|KIJ69967.1|  hypothetical protein HYDPIDRAFT_78947 [Hydnomerul...  38.9    4.5   
gb|KIK06824.1|  hypothetical protein K443DRAFT_129744 [Laccaria a...  38.9    5.0   
gb|KIJ20368.1|  hypothetical protein PAXINDRAFT_108374 [Paxillus ...  38.9    5.1   
ref|XP_001876618.1|  predicted protein [Laccaria bicolor S238N-H8...  38.5    6.2   
ref|WP_051781152.1|  thiamine biosynthesis protein ThiJ [Janthino...  38.1    7.1   
gb|KIY73842.1|  hypothetical protein CYLTODRAFT_385849 [Cylindrob...  38.1    9.3   


 >emb|CDZ98239.1| hypothetical protein [Xanthophyllomyces dendrorhous]
Length=284

 Score =  467 bits (1201),  Expect = 4e-163, Method: Compositional matrix adjust.
 Identities = 274/284 (96%), Positives = 274/284 (96%), Gaps = 10/284 (4%)

Query  1    MNVPLPILPPRDYPVTRFASSPLPRFLRTVTFLLSLLLGSSAIAAYLYTKIILPRLIRSR  60
            MNVPLPILPPRDYPVTRFASSPLPRFLRTVTFLLSLLLGSSAIAAYLYTKIILPRLIRSR
Sbjct  1    MNVPLPILPPRDYPVTRFASSPLPRFLRTVTFLLSLLLGSSAIAAYLYTKIILPRLIRSR  60

Query  61   EARDILLQKHVSHLDSLRISLNRYLDVGFVRNSPSNALPTDNLLIEGSQNEIEASLAADS  120
            EARDILLQKHVSHLDSLRISLNRYLDVGFVRNSPSNALPTDNLLIEGSQNEIEASLAADS
Sbjct  61   EARDILLQKHVSHLDSLRISLNRYLDVGFVRNSPSNALPTDNLLIEGSQNEIEASLAADS  120

Query  121  LTQPLVSSVKEAETEIAPSDTLTIEASSGKDESSLIRADSVLGLSVEKDPVGLPMDDLGD  180
            LTQPLVSSVKEAETEIAPSDTLTIEASSGKDESSLIRADSVLGLSVEKDPVGLPMDDLGD
Sbjct  121  LTQPLVSSVKEAETEIAPSDTLTIEASSGKDESSLIRADSVLGLSVEKDPVGLPMDDLGD  180

Query  181  LYALLLMLAPKERPTGLLRAIKGAGASIVSQSLLFNSKT----------LSNQLERAGAV  230
            LYALLLMLAPKERPTGLLRAIKGAGASIVSQSLLFNSKT          LSNQLERAGAV
Sbjct  181  LYALLLMLAPKERPTGLLRAIKGAGASIVSQSLLFNSKTTRVSSNGSYSLSNQLERAGAV  240

Query  231  GSGGAWGGGLGPGEREVISLIGGLRGDVRGFKGALLSRRALSNP  274
            GSGGAWGGGLGPGEREVISLIGGLRGDVRGFKGALLSRRALSNP
Sbjct  241  GSGGAWGGGLGPGEREVISLIGGLRGDVRGFKGALLSRRALSNP  284


>gb|ETS64000.1| hypothetical protein PaG_02336 [Pseudozyma aphidis DSM 70725]
Length=568

 Score = 48.9 bits (115),  Expect = 0.003, Method: Compositional matrix adjust.
 Identities = 36/125 (29%), Positives = 61/125 (49%), Gaps = 11/125 (9%)

Query  14   PVTRFASSPLPRFLRTVTFLLSLLLGSSAIAAYLYTKIILPRLIRSREARDILLQKHVSH  73
            P+TR+    L +F RT+++++ L  G +AI  + Y   ILP++    +AR I+L+ H+  
Sbjct  188  PLTRY--QVLLQFFRTLSYMMMLGGGLTAIVVWAYRAYILPKITAMFDARSIVLKHHIML  245

Query  74   LDSLR---ISLNRYLDVGFVRNSPSNALPTDNLLIEGSQNEIEASLAADSLTQPLVSSVK  130
             D LR    SL  Y+ V   R      +  D+    G+ + ++  L        + +S K
Sbjct  246  FDKLRSNVASLATYVPVQRAR------IGGDDASSNGASSPLKGVLKKVHFHDDVDTSAK  299

Query  131  EAETE  135
            E E E
Sbjct  300  EGEKE  304


>dbj|GAC74011.1| hypothetical protein PANT_9c00408 [Pseudozyma antarctica T-34]
Length=533

 Score = 48.1 bits (113),  Expect = 0.005, Method: Compositional matrix adjust.
 Identities = 39/142 (27%), Positives = 66/142 (46%), Gaps = 11/142 (8%)

Query  14   PVTRFASSPLPRFLRTVTFLLSLLLGSSAIAAYLYTKIILPRLIRSREARDILLQKHVSH  73
            P+TR+    L +F RT+++++ L  G +AI    Y   ILP++    +AR I+L+ H+  
Sbjct  153  PLTRY--QVLLQFFRTLSYMMMLGGGLTAIVVSAYRAYILPKITAMFDARSIVLKHHIML  210

Query  74   LDSLR---ISLNRYLDVGFVRNSPSNALPTDNLLIEGSQNEIEASLAADSLTQPLVSSVK  130
             D LR    SL  Y+ V   R      +  D+    G+ + ++  L        + +S K
Sbjct  211  FDKLRSNVASLATYVPVQRAR------IGGDDASTNGASSPLKGVLKKVHFHDDVDTSAK  264

Query  131  EAETEIAPSDTLTIEASSGKDE  152
            E E E         +A +G D+
Sbjct  265  EGEKEGVKLTQHAKDAKAGSDK  286


>gb|KIY49436.1| hypothetical protein FISHEDRAFT_41153 [Fistulina hepatica ATCC 
64428]
Length=424

 Score = 45.8 bits (107),  Expect = 0.027, Method: Compositional matrix adjust.
 Identities = 28/77 (36%), Positives = 41/77 (53%), Gaps = 2/77 (3%)

Query  6    PILPPRDYPVTRFASSPLPRFLRTVTFLLSLLLGSSAIAAYLYTKIILPRLIRSREARDI  65
            P +PPR YP  +   S LP  L  +  L S +LGS++I   +Y   +LPR+I++  AR  
Sbjct  120  PTIPPRTYP--QLPPSNLPNLLYKIARLFSWILGSASIVIAIYYYFLLPRIIKTAVARKA  177

Query  66   LLQKHVSHLDSLRISLN  82
            L     + L  L  SL+
Sbjct  178  LNSHQANLLRRLTESLH  194


>emb|CBQ68180.1| conserved hypothetical protein [Sporisorium reilianum SRZ2]
Length=521

 Score = 45.8 bits (107),  Expect = 0.029, Method: Compositional matrix adjust.
 Identities = 27/81 (33%), Positives = 45/81 (56%), Gaps = 5/81 (6%)

Query  14   PVTRFASSPLPRFLRTVTFLLSLLLGSSAIAAYLYTKIILPRLIRSREARDILLQKHVSH  73
            P+TR+    L +F RT++FL+ L  G++A+    Y   +LP++    +AR I+L+ H+  
Sbjct  149  PLTRYQV--LLQFFRTLSFLMMLSGGATALVVSAYRAYVLPKITAMFDARSIVLKHHIVL  206

Query  74   LDSLRIS---LNRYLDVGFVR  91
             D LR +   L  Y+ V   R
Sbjct  207  FDKLRSNVAGLATYVPVQHAR  227


>gb|KNZ77176.1| hypothetical protein J132_06171 [Termitomyces sp. J132]
Length=423

 Score = 45.4 bits (106),  Expect = 0.036, Method: Compositional matrix adjust.
 Identities = 28/76 (37%), Positives = 41/76 (54%), Gaps = 2/76 (3%)

Query  6    PILPPRDYPVTRFASSPLPRFLRTVTFLLSLLLGSSAIAAYLYTKIILPRLIRSREARDI  65
            P +PPR YP  +   S LP  L  ++ + S ++G S    ++Y +  LPR+I+S  AR  
Sbjct  112  PAIPPRAYP--QLPPSNLPTLLLGLSRVFSWIVGGSTALIFIYYRFFLPRIIKSSLARQS  169

Query  66   LLQKHVSHLDSLRISL  81
            L   H+S L  L  SL
Sbjct  170  LKAHHLSLLRKLNESL  185


>dbj|GAK61885.1| conserved hypothetical protein [Pseudozyma antarctica]
Length=533

 Score = 45.1 bits (105),  Expect = 0.060, Method: Compositional matrix adjust.
 Identities = 28/81 (35%), Positives = 44/81 (54%), Gaps = 5/81 (6%)

Query  14   PVTRFASSPLPRFLRTVTFLLSLLLGSSAIAAYLYTKIILPRLIRSREARDILLQKHVSH  73
            P+TR+    L +F RT+++++ L  G +AI    Y   ILP++    +AR I+L+ H+  
Sbjct  153  PLTRY--QVLLQFFRTLSYMMMLGGGLTAIVVSAYRAYILPKITAMFDARSIVLKHHIML  210

Query  74   LDSLR---ISLNRYLDVGFVR  91
             D LR    SL  Y+ V   R
Sbjct  211  FDKLRSNVASLATYVPVQRAR  231


>gb|EST06686.1| hypothetical protein PSEUBRA_SCAF3g04206 [Pseudozyma brasiliensis 
GHG001]
Length=515

 Score = 44.7 bits (104),  Expect = 0.076, Method: Compositional matrix adjust.
 Identities = 28/81 (35%), Positives = 44/81 (54%), Gaps = 5/81 (6%)

Query  14   PVTRFASSPLPRFLRTVTFLLSLLLGSSAIAAYLYTKIILPRLIRSREARDILLQKHVSH  73
            P+TR+    L +F RT++F++ L  G +A+    Y   ILP++    +AR I+L+ H+  
Sbjct  154  PLTRYQV--LLQFFRTLSFMMMLGGGLTALLVSAYRSYILPKITAMFDARSIVLKHHIVL  211

Query  74   LDSLR---ISLNRYLDVGFVR  91
             D LR    SL  Y+ V   R
Sbjct  212  FDKLRSNVASLATYVPVQHAR  232


>gb|EUC66862.1| flavin containing monooxygenase FMO GS-OX3 [Rhizoctonia solani 
AG-3 Rhs1AP]
 gb|KEP55125.1| flavin containing monooxygenase FMO GS-OX3 [Rhizoctonia solani 
123E]
Length=312

 Score = 44.3 bits (103),  Expect = 0.087, Method: Compositional matrix adjust.
 Identities = 25/84 (30%), Positives = 45/84 (54%), Gaps = 2/84 (2%)

Query  5    LPILPPRDYPVTRFA--SSPLPRFLRTVTFLLSLLLGSSAIAAYLYTKIILPRLIRSREA  62
            +PI+PPR YP T  +   S L   L T+ +  +   G+SA+  ++Y+K + PR ++   A
Sbjct  103  IPIVPPRTYPETLVSPTRSRLFENLVTIYYFFTYAAGASAVLTWVYSKFVFPRWVKMILA  162

Query  63   RDILLQKHVSHLDSLRISLNRYLD  86
            +  L +  +  L+ L  +L  + D
Sbjct  163  KRRLREHQLGLLEHLTENLREHRD  186


>ref|XP_007315756.1| hypothetical protein SERLADRAFT_446905 [Serpula lacrymans var. 
lacrymans S7.9]
 gb|EGO27665.1| hypothetical protein SERLADRAFT_446905 [Serpula lacrymans var. 
lacrymans S7.9]
Length=399

 Score = 43.5 bits (101),  Expect = 0.14, Method: Compositional matrix adjust.
 Identities = 38/132 (29%), Positives = 63/132 (48%), Gaps = 11/132 (8%)

Query  11   RDYPVTRFASSPLPRFLRTVTFLLSLLLGSSAIAAYLYTKIILPRLIRSREARDILLQKH  70
            R YP  + A S LP  L  +  + + + G+SA   ++Y + ILPR+ R+ EAR +L++  
Sbjct  82   RTYP--QPAPSNLPNLLIGLARIFTWITGTSAALLFIYYRFILPRITRTYEARHMLIKHQ  139

Query  71   VSHLDSLRISLNRYLDVGFVRNSPSNALPTDNLLIEGSQNEIEASLAADSLTQPLVSSVK  130
             S L     SL    D    + S    LP  +   E S+     +L A      L++++ 
Sbjct  140  SSLLSRFTDSLRTLKD---TQESSYVELPRKSHPAEDSRYAQHHTLEA------LLAAIG  190

Query  131  EAETEIAPSDTL  142
            + + E  P +TL
Sbjct  191  DGDPESIPDETL  202


>emb|CDS01462.1| hypothetical protein [Sporisorium scitamineum]
Length=519

 Score = 43.9 bits (102),  Expect = 0.14, Method: Compositional matrix adjust.
 Identities = 27/81 (33%), Positives = 44/81 (54%), Gaps = 5/81 (6%)

Query  14   PVTRFASSPLPRFLRTVTFLLSLLLGSSAIAAYLYTKIILPRLIRSREARDILLQKHVSH  73
            P+TR+    L +F RTV++++ L  G +A+    Y   ILP++    +AR I+L+ H+  
Sbjct  156  PLTRYQV--LLQFFRTVSYMMMLGGGLTALLVSAYRAYILPKITAMFDARSIVLKHHIVL  213

Query  74   LDSLR---ISLNRYLDVGFVR  91
             D LR    SL  Y+ +   R
Sbjct  214  FDRLRSNVASLATYVPIQHTR  234


>gb|EGO02042.1| hypothetical protein SERLA73DRAFT_166545 [Serpula lacrymans var. 
lacrymans S7.3]
Length=377

 Score = 43.5 bits (101),  Expect = 0.15, Method: Compositional matrix adjust.
 Identities = 38/132 (29%), Positives = 63/132 (48%), Gaps = 11/132 (8%)

Query  11   RDYPVTRFASSPLPRFLRTVTFLLSLLLGSSAIAAYLYTKIILPRLIRSREARDILLQKH  70
            R YP  + A S LP  L  +  + + + G+SA   ++Y + ILPR+ R+ EAR +L++  
Sbjct  82   RTYP--QPAPSNLPNLLIGLARIFTWITGTSAALLFIYYRFILPRITRTYEARHMLIKHQ  139

Query  71   VSHLDSLRISLNRYLDVGFVRNSPSNALPTDNLLIEGSQNEIEASLAADSLTQPLVSSVK  130
             S L     SL    D    + S    LP  +   E S+     +L A      L++++ 
Sbjct  140  SSLLSRFTDSLRTLKD---TQESSYVELPRKSHPAEDSRYAQHHTLEA------LLAAIG  190

Query  131  EAETEIAPSDTL  142
            + + E  P +TL
Sbjct  191  DGDPESIPDETL  202


>ref|XP_007268209.1| hypothetical protein FOMMEDRAFT_147624, partial [Fomitiporia 
mediterranea MF3/22]
 gb|EJD00932.1| hypothetical protein FOMMEDRAFT_147624, partial [Fomitiporia 
mediterranea MF3/22]
Length=345

 Score = 43.5 bits (101),  Expect = 0.15, Method: Compositional matrix adjust.
 Identities = 26/61 (43%), Positives = 35/61 (57%), Gaps = 0/61 (0%)

Query  21   SPLPRFLRTVTFLLSLLLGSSAIAAYLYTKIILPRLIRSREARDILLQKHVSHLDSLRIS  80
            S LP  L  +T + + + GSSA+  ++Y K +LPRL RS  AR  L Q  V+ L  L  S
Sbjct  140  SKLPYLLLGLTRIFTWITGSSAVLIFIYYKFLLPRLTRSALARRALKQHQVALLKRLNAS  199

Query  81   L  81
            L
Sbjct  200  L  200


>ref|XP_013240274.1| hypothetical protein K437DRAFT_259903 [Tilletiaria anomala UBC 
951]
 gb|KDN37209.1| hypothetical protein K437DRAFT_259903 [Tilletiaria anomala UBC 
951]
Length=644

 Score = 43.5 bits (101),  Expect = 0.17, Method: Compositional matrix adjust.
 Identities = 24/57 (42%), Positives = 36/57 (63%), Gaps = 2/57 (4%)

Query  14   PVTRFASSPLPRFLRTVTFLLSLLLGSSAIAAYLYTKIILPRLIRSREARDILLQKH  70
            P+TR+    L  F R +++L+ L  G +A+   LY   I+PRLI S++AR ILL+ H
Sbjct  187  PLTRY--QVLLNFFRQLSYLMMLGGGFTALLVGLYRIYIMPRLIASQDARSILLKHH  241


>gb|KDQ64378.1| hypothetical protein JAAARDRAFT_166529 [Jaapia argillacea MUCL 
33604]
Length=386

 Score = 43.1 bits (100),  Expect = 0.21, Method: Compositional matrix adjust.
 Identities = 25/72 (35%), Positives = 37/72 (51%), Gaps = 2/72 (3%)

Query  11   RDYPVTRFASSPLPRFLRTVTFLLSLLLGSSAIAAYLYTKIILPRLIRSREARDILLQKH  70
            R YP  +   S LP  L  +  + S + G SA+  ++Y + +LPRL RS +AR  L   H
Sbjct  91   RTYP--QPTPSNLPNLLVGIARIFSWVAGGSAVVLFMYYRFLLPRLTRSFQARHALRMHH  148

Query  71   VSHLDSLRISLN  82
               ++ L  SL 
Sbjct  149  TGLMERLNTSLT  160


>ref|XP_012193108.1| hypothetical protein PHSY_007123 [Pseudozyma hubeiensis SY62]
 dbj|GAC99521.1| hypothetical protein PHSY_007123 [Pseudozyma hubeiensis SY62]
Length=711

 Score = 43.1 bits (100),  Expect = 0.26, Method: Compositional matrix adjust.
 Identities = 28/81 (35%), Positives = 44/81 (54%), Gaps = 5/81 (6%)

Query  14   PVTRFASSPLPRFLRTVTFLLSLLLGSSAIAAYLYTKIILPRLIRSREARDILLQKHVSH  73
            P+TR+    L +F RT+++++ L  G +AI    Y   ILP++    +AR I+L+ H+  
Sbjct  149  PLTRYQV--LLQFFRTLSYMMMLSGGLTAILVSSYRAYILPKITAMFDARSIVLKHHIVL  206

Query  74   LDSLRIS---LNRYLDVGFVR  91
             D LR S   L  Y+ V   R
Sbjct  207  FDRLRSSVAGLATYVPVQHAR  227


>ref|XP_002911173.1| hypothetical protein CC1G_14604 [Coprinopsis cinerea okayama7#130]
 gb|EFI27679.1| hypothetical protein CC1G_14604 [Coprinopsis cinerea okayama7#130]
Length=403

 Score = 42.7 bits (99),  Expect = 0.28, Method: Compositional matrix adjust.
 Identities = 26/76 (34%), Positives = 40/76 (53%), Gaps = 2/76 (3%)

Query  6    PILPPRDYPVTRFASSPLPRFLRTVTFLLSLLLGSSAIAAYLYTKIILPRLIRSREARDI  65
            P++PPR YP  +   S LP  L  +  L S L   S    ++Y + +LP++ R+ +AR  
Sbjct  97   PVIPPRTYP--QAPPSNLPTLLLGLARLFSWLGMGSVALIFVYYRFLLPKITRTAQARHS  154

Query  66   LLQKHVSHLDSLRISL  81
            L   H+S +  L  SL
Sbjct  155  LRTHHISLMQKLTASL  170


>ref|XP_011386534.1| hypothetical protein UMAG_00755 [Ustilago maydis 521]
 gb|KIS72350.1| hypothetical protein UMAG_00755 [Ustilago maydis 521]
Length=544

 Score = 42.7 bits (99),  Expect = 0.29, Method: Compositional matrix adjust.
 Identities = 27/81 (33%), Positives = 42/81 (52%), Gaps = 5/81 (6%)

Query  14   PVTRFASSPLPRFLRTVTFLLSLLLGSSAIAAYLYTKIILPRLIRSREARDILLQKHVSH  73
            P+TR+    L +F RT+++++ L  G +AI    Y   ILP++     AR I+L+ H+  
Sbjct  160  PLTRY--QVLLQFFRTLSYMMMLSGGLTAIVVSSYRAYILPKITAMFHARSIVLKHHIVL  217

Query  74   LDSLR---ISLNRYLDVGFVR  91
             D LR    SL  Y  +   R
Sbjct  218  FDKLRSNVASLAMYAPIQHAR  238


>ref|XP_007879121.1| hypothetical protein PFL1_03413 [Pseudozyma flocculosa PF-1]
 gb|EPQ29125.1| hypothetical protein PFL1_03413 [Pseudozyma flocculosa PF-1]
Length=577

 Score = 42.7 bits (99),  Expect = 0.29, Method: Compositional matrix adjust.
 Identities = 23/68 (34%), Positives = 41/68 (60%), Gaps = 2/68 (3%)

Query  14   PVTRFASSPLPRFLRTVTFLLSLLLGSSAIAAYLYTKIILPRLIRSREARDILLQKHVSH  73
            P+TR+    L +F +T+++L+ L  G +A++  L+   +LPR+  + +AR I+L  H   
Sbjct  172  PLTRYQV--LLQFFKTLSYLMMLGGGVTALSVALWRTYVLPRITATFDARSIVLSHHHVL  229

Query  74   LDSLRISL  81
             D LR S+
Sbjct  230  YDKLRASV  237


>ref|XP_007768011.1| hypothetical protein CONPUDRAFT_164918 [Coniophora puteana RWD-64-598 
SS2]
 gb|EIW82296.1| hypothetical protein CONPUDRAFT_164918 [Coniophora puteana RWD-64-598 
SS2]
Length=423

 Score = 42.0 bits (97),  Expect = 0.45, Method: Compositional matrix adjust.
 Identities = 23/65 (35%), Positives = 33/65 (51%), Gaps = 0/65 (0%)

Query  23   LPRFLRTVTFLLSLLLGSSAIAAYLYTKIILPRLIRSREARDILLQKHVSHLDSLRISLN  82
            LP  L  V+ ++S   G+SA+  ++Y + ILPRL  S +AR  +       L  L  SL 
Sbjct  103  LPNLLIGVSRIVSWFFGTSAVLVFIYYRYILPRLTHSYQARHSIQSHQTDLLSRLNSSLA  162

Query  83   RYLDV  87
             Y  V
Sbjct  163  AYKSV  167


>emb|CUA76679.1| hypothetical protein RSOLAG22IIIB_02146 [Rhizoctonia solani]
Length=318

 Score = 41.6 bits (96),  Expect = 0.65, Method: Compositional matrix adjust.
 Identities = 24/82 (29%), Positives = 41/82 (50%), Gaps = 2/82 (2%)

Query  5    LPILPPRDYP--VTRFASSPLPRFLRTVTFLLSLLLGSSAIAAYLYTKIILPRLIRSREA  62
            +PI+PPR YP  +     S L   L TV +  +   G+SA+  ++Y+K + PR ++   A
Sbjct  107  IPIVPPRTYPEALVSPTRSRLFESLVTVYYFFTYAAGASAVLTWVYSKFVFPRWVKMITA  166

Query  63   RDILLQKHVSHLDSLRISLNRY  84
            +  L +     L  L   L+ +
Sbjct  167  KRRLREHQFGLLKRLTEDLSEH  188


>gb|KIM68921.1| hypothetical protein SCLCIDRAFT_1209138 [Scleroderma citrinum 
Foug A]
Length=467

 Score = 41.6 bits (96),  Expect = 0.66, Method: Compositional matrix adjust.
 Identities = 25/62 (40%), Positives = 36/62 (58%), Gaps = 0/62 (0%)

Query  21   SPLPRFLRTVTFLLSLLLGSSAIAAYLYTKIILPRLIRSREARDILLQKHVSHLDSLRIS  80
            S LP  L  V  +L+ L GSSAI   +Y + +LPR+ +S  AR  L   H+S L+ L+ S
Sbjct  96   SALPNLLIGVARVLTWLTGSSAIILLIYYRFLLPRISQSYHARHALRSHHLSLLERLKNS  155

Query  81   LN  82
             +
Sbjct  156  AH  157


>emb|CCF51250.1| uncharacterized protein UHOR_01156 [Ustilago hordei]
Length=547

 Score = 41.2 bits (95),  Expect = 1.0, Method: Compositional matrix adjust.
 Identities = 27/81 (33%), Positives = 44/81 (54%), Gaps = 5/81 (6%)

Query  14   PVTRFASSPLPRFLRTVTFLLSLLLGSSAIAAYLYTKIILPRLIRSREARDILLQKHVSH  73
            P+TR+    L +F RT+++++ L  G +AI    +   ILP++    +AR I+L+ H+  
Sbjct  157  PLTRYQV--LLQFFRTLSYMMMLGGGLTAILVSAFRAYILPKITAMFDARSIVLKHHIVL  214

Query  74   LDSLR---ISLNRYLDVGFVR  91
             D LR    SL  Y+ V   R
Sbjct  215  FDKLRSNVASLATYVPVQPAR  235


>gb|KDE09189.1| hypothetical protein MVLG_00511 [Microbotryum violaceum p1A1 
Lamole]
Length=382

 Score = 40.8 bits (94),  Expect = 1.2, Method: Compositional matrix adjust.
 Identities = 29/91 (32%), Positives = 43/91 (47%), Gaps = 22/91 (24%)

Query  3    VPLPILPP---RDY------------------PVTRFASSPLPRFLRTVTFLLSLLLGSS  41
             P P LPP   RDY                  P     +S +P F+RT+  L+ ++ G+S
Sbjct  49   TPTPSLPPIGPRDYNNPTSSSLSSFSIRPVNLPPAYAPNSTVPTFIRTLAILVFVVGGTS  108

Query  42   AIA-AYLYTKIILPRLIRSREARDILLQKHV  71
            A   A++Y  I+ PRL+ +  AR  L Q H+
Sbjct  109  AATIAWVYKNIVYPRLVLALRARTTLHQGHL  139


>emb|CDI51690.1| putative protein [Melanopsichium pennsylvanicum 4]
Length=534

 Score = 40.4 bits (93),  Expect = 1.5, Method: Compositional matrix adjust.
 Identities = 20/54 (37%), Positives = 32/54 (59%), Gaps = 0/54 (0%)

Query  25   RFLRTVTFLLSLLLGSSAIAAYLYTKIILPRLIRSREARDILLQKHVSHLDSLR  78
            +F RT+++L+ L  G +AI    Y   ILP++    +AR I+L+ H+   D LR
Sbjct  159  QFFRTLSYLMMLSGGLTAILVGSYRAYILPKITAMFDARSIVLKHHIVLFDKLR  212


>gb|EMS23907.1| peroxisomal membrane anchor protein [Rhodosporidium toruloides 
NP11]
 emb|CDR40619.1| RHTO0S05e05534g1_1 [Rhodosporidium toruloides]
Length=326

 Score = 40.0 bits (92),  Expect = 1.7, Method: Compositional matrix adjust.
 Identities = 30/89 (34%), Positives = 44/89 (49%), Gaps = 9/89 (10%)

Query  4    PLPILPPRDY--------PVTRFASSPLPRFLRTVTFLLSLLLGSSAIAAYLYTKIILPR  55
            P P+ PPR Y        P     +S +P F+RT++ LL +    SA  A++Y  II PR
Sbjct  37   PAPV-PPRTYSQLPIYAPPPAYAPNSTVPTFIRTLSILLFVGGTLSAAVAWVYKSIIYPR  95

Query  56   LIRSREARDILLQKHVSHLDSLRISLNRY  84
            L+ + +AR  L   H S    L  +L  +
Sbjct  96   LVVALKARSRLFTIHESAYAKLYDALRTF  124


>emb|CCO26838.1| Flavin-containing monooxygenase FMO GS-OX3 [Rhizoctonia solani 
AG-1 IB]
Length=705

 Score = 40.0 bits (92),  Expect = 2.1, Method: Compositional matrix adjust.
 Identities = 22/82 (27%), Positives = 42/82 (51%), Gaps = 2/82 (2%)

Query  5    LPILPPRDYPVTRFASSPLPRF--LRTVTFLLSLLLGSSAIAAYLYTKIILPRLIRSREA  62
            +P++PPR YP    + +    F  L T+ +  +   G+SA+  ++Y+K + PR ++   A
Sbjct  110  IPVVPPRTYPEALVSPTRSRLFDNLVTIYYFFTYAAGASAVLTWVYSKFVFPRWVKMIMA  169

Query  63   RDILLQKHVSHLDSLRISLNRY  84
            +  L Q  +  L  L   L+ +
Sbjct  170  KRRLRQHQLGLLKRLTEELSDH  191


>emb|CEL57668.1| hypothetical protein RSOLAG1IB_02411 [Rhizoctonia solani AG-1 
IB]
Length=316

 Score = 39.3 bits (90),  Expect = 2.8, Method: Compositional matrix adjust.
 Identities = 22/82 (27%), Positives = 42/82 (51%), Gaps = 2/82 (2%)

Query  5    LPILPPRDYPVTRFASSPLPRF--LRTVTFLLSLLLGSSAIAAYLYTKIILPRLIRSREA  62
            +P++PPR YP    + +    F  L T+ +  +   G+SA+  ++Y+K + PR ++   A
Sbjct  110  IPVVPPRTYPEALVSPTRSRLFDNLVTIYYFFTYAAGASAVLTWVYSKFVFPRWVKMIMA  169

Query  63   RDILLQKHVSHLDSLRISLNRY  84
            +  L Q  +  L  L   L+ +
Sbjct  170  KRRLRQHQLGLLKRLTEELSDH  191


>emb|CDY65871.1| BnaAnng21200D [Brassica napus]
Length=457

 Score = 39.3 bits (90),  Expect = 4.1, Method: Compositional matrix adjust.
 Identities = 42/142 (30%), Positives = 67/142 (47%), Gaps = 13/142 (9%)

Query  122  TQPLVSSVKEAETEIAPSDTLTIEASSGKDESSLIRADSV-------LGLSVEKDPVG--  172
            TQ LV +V+E +  +  S  LT+ A++G +   +++           L L V +   G  
Sbjct  100  TQLLVGAVREKDRIMWESICLTLAATAGNNIGKVLQKKGTIILPPLSLKLKVIRAYAGNK  159

Query  173  -LPMDDLGDLYALLLML-APKERPTGLLRAIKGAGASIVSQSLLFNSKTLSNQLERAGAV  230
               +  L D++  LLML A    P  +++ + G G +I+S    F  K + N  +  G  
Sbjct  160  PWALGFLMDIFGALLMLRALSLAPVSVVQPVSGCGLAILSVFSHFYLKEVMNVFDWIGIT  219

Query  231  GSG-GAWGGGLGPGEREVISLI  251
             +G G  G G G GE +V SLI
Sbjct  220  MAGIGTIGVGAG-GEEQVASLI  240


>gb|KIJ69967.1| hypothetical protein HYDPIDRAFT_78947 [Hydnomerulius pinastri 
MD-312]
Length=420

 Score = 38.9 bits (89),  Expect = 4.5, Method: Compositional matrix adjust.
 Identities = 21/54 (39%), Positives = 33/54 (61%), Gaps = 1/54 (2%)

Query  21   SPLPRFLRTVTFLLSLLLGSSAIAAYLYTKIILPRLIRSREARDILLQKHVSHL  74
            S LP  L  +  + + L G+SA+  ++Y + +LPRL RS +AR + L+ H S L
Sbjct  100  SNLPNLLIGIARIFTWLTGTSAVILFIYYRYLLPRLTRSYQAR-LALRNHQSTL  152


>gb|KIK06824.1| hypothetical protein K443DRAFT_129744 [Laccaria amethystina LaAM-08-1]
Length=394

 Score = 38.9 bits (89),  Expect = 5.0, Method: Compositional matrix adjust.
 Identities = 23/59 (39%), Positives = 33/59 (56%), Gaps = 0/59 (0%)

Query  23   LPRFLRTVTFLLSLLLGSSAIAAYLYTKIILPRLIRSREARDILLQKHVSHLDSLRISL  81
            LP  L  ++ L S L G SA   ++Y + +LPR+ +S  AR  L   H+S L +L  SL
Sbjct  92   LPNLLLGISRLFSWLAGGSATLIFIYYRFLLPRITKSLMARQSLKAHHLSLLRNLTTSL  150


>gb|KIJ20368.1| hypothetical protein PAXINDRAFT_108374 [Paxillus involutus ATCC 
200175]
Length=426

 Score = 38.9 bits (89),  Expect = 5.1, Method: Compositional matrix adjust.
 Identities = 22/54 (41%), Positives = 32/54 (59%), Gaps = 1/54 (2%)

Query  21   SPLPRFLRTVTFLLSLLLGSSAIAAYLYTKIILPRLIRSREARDILLQKHVSHL  74
            S LP     V  + + L G+SA+A ++Y   +LPRL +S +AR + L KH S L
Sbjct  105  SNLPNLFIGVARIFTWLTGTSAVALFIYYWYLLPRLTQSYQAR-LALSKHQSGL  157


>ref|XP_001876618.1| predicted protein [Laccaria bicolor S238N-H82]
 gb|EDR12354.1| predicted protein [Laccaria bicolor S238N-H82]
Length=392

 Score = 38.5 bits (88),  Expect = 6.2, Method: Compositional matrix adjust.
 Identities = 24/61 (39%), Positives = 33/61 (54%), Gaps = 0/61 (0%)

Query  21   SPLPRFLRTVTFLLSLLLGSSAIAAYLYTKIILPRLIRSREARDILLQKHVSHLDSLRIS  80
            S LP  L  +  L S L G SA   ++Y + +LPR+ +S  AR  L   H+S L +L  S
Sbjct  90   SNLPNLLLGICRLFSWLAGGSAALIFIYYRFLLPRITQSFMARQSLKAHHLSLLRNLTTS  149

Query  81   L  81
            L
Sbjct  150  L  150


>ref|WP_051781152.1| thiamine biosynthesis protein ThiJ [Janthinobacterium agaricidamnosum]
 emb|CDG84848.1| DJ-1/PfpI family protein [Janthinobacterium agaricidamnosum NBRC 
102515 = DSM 9628]
Length=303

 Score = 38.1 bits (87),  Expect = 7.1, Method: Compositional matrix adjust.
 Identities = 43/178 (24%), Positives = 74/178 (42%), Gaps = 34/178 (19%)

Query  73   HLDSLRISLNRYLDVGF--VRNSPSNALPT------DNLLIEGSQNEIEASLAADSLTQP  124
            +LD L + L + LD G+  V  SP+   P+      D +  EGS+ ++ A+         
Sbjct  66   YLDELALPLRKLLDAGYTPVFASPNGTAPSYDQVSHDKMFFEGSEEKLAAA---------  116

Query  125  LVSSVKEAETEIAPSDTLTIEASSGKDESSLIRADSVLGLSVEKDPVG-LPMDDLG---D  180
             V  V   +  + P     + A   K+          +G+ +   P G  PM DL    D
Sbjct  117  -VKLVNSEQGPLHPKKLSAVLAEGTKN---------YVGIFI---PGGHAPMQDLSQDKD  163

Query  181  LYALLLMLAPKERPTGLLRAIKGAGASIVSQSLLFNSKTLSNQLERAGAVGSGGAWGG  238
            L  +LL     +RPTG++     A  S +S  + F    ++  ++  G + +G  + G
Sbjct  164  LGKILLTFHDTQRPTGIICHGPTALLSTLSDPVAFRQALIAGDIQSTGKLAAGWPYAG  221


>gb|KIY73842.1| hypothetical protein CYLTODRAFT_385849 [Cylindrobasidium torrendii 
FP15055 ss-10]
Length=401

 Score = 38.1 bits (87),  Expect = 9.3, Method: Compositional matrix adjust.
 Identities = 21/61 (34%), Positives = 35/61 (57%), Gaps = 0/61 (0%)

Query  21   SPLPRFLRTVTFLLSLLLGSSAIAAYLYTKIILPRLIRSREARDILLQKHVSHLDSLRIS  80
            S LP  L  V  + S   G++A+  ++Y + +LPR+ ++ EAR  L  +H+S +  L  S
Sbjct  96   SNLPTMLLGVLRIFSWATGATALLVFVYYRYLLPRITQTSEARHNLRTQHLSLMRRLTTS  155

Query  81   L  81
            L
Sbjct  156  L  156


Lambda      K        H        a         alpha
   0.318    0.137    0.383    0.792     4.96 

Gapped
Lambda      K        H        a         alpha    sigma
   0.267   0.0410    0.140     1.90     42.6     43.6 

Effective search space used: 1963939664823


  Database: nr
    Posted date:  Sep 23, 2015 12:05 AM
  Number of letters in database: 26,053,659,533
  Number of sequences in database:  71,551,133


Matrix: BLOSUM62
Gap Penalties: Existence: 11, Extension: 1
Neighboring words threshold: 11
Window for multiple hits: 40
```
